# Effects of Probiotic Bacteria *Lactobacillaceae* on the Gut Microbiota in Children With Celiac Disease Autoimmunity: A Placebo-Controlled and Randomized Clinical Trial

**DOI:** 10.3389/fnut.2021.680771

**Published:** 2021-06-25

**Authors:** Elin Oscarsson, Åsa Håkansson, Carin Andrén Aronsson, Göran Molin, Daniel Agardh

**Affiliations:** ^1^The Diabetes and Celiac Disease Unit, Department of Clinical Sciences, Lund University, Malmö, Sweden; ^2^Department of Food Technology Engineering and Nutrition, Lund University, Lund, Sweden

**Keywords:** celiac disease, probiotic, *Lactobacillaceae*, autoimmunity, gut microbiota

## Abstract

Disturbances of the gut microbiota may influence the development of various autoimmune diseases. This study investigated the effects of supplementations with the probiotic bacteria, *Lactiplantibacillus plantarum* HEAL9 and *Lacticaseibacillus paracasei* 8700:2, on the microbial community in children with celiac disease autoimmunity (CDA). The study included 78 genetically predisposed children for celiac disease with elevated levels of tissue transglutaminase autoantibodies (tTGA) signaling for ongoing CDA. Among those children, 38 received a placebo and 40 received the probiotic supplement daily for 6 months. Fecal and plasma samples were collected at baseline and after 3 and 6 months, respectively. The bacterial community was investigated with 16S rRNA gene sequencing and terminal restriction fragment length polymorphism (T-RFLP), and tTGA levels were measured in radiobinding assays. In children that received probiotic supplementation, the relative abundance of *Lactobacillaceae* increased over time, while it remained unchanged in the placebo group. There was no overall correlation between tTGA levels and bacterial genus except for a positive correlation between *Dialister* and IgG-tTG in the probiotic group. The abundance of specific bacterial amplicon sequence variant (ASV:s) changed during the study in both groups, indicating that specific bacterial strains might be affected by probiotic supplementation.

## Introduction

Celiac disease is characterized by a damaged intestinal mucosa barrier caused by an immune-mediated response after ingestion of gluten in individuals carrying either the human leukocyte antigen (HLA) haplotypes DQA1^*^05:01-DQB1^*^02:01 (abbreviated DQ2) and/or DQA1^*^03:01-DQB1^*^03:02 (abbreviated DQ8) (1). There are indications that HLA-DQ2 carriers have different bacterial colonization of the gut compared with individuals not carrying this at-risk haplotype for celiac disease (2, 3). At diagnosis, individuals with celiac disease have an imbalance of the gut microbiota composition that only partially returns to normal after years on a gluten-free diet (4). In addition, patients with celiac disease treated with a gluten-free diet have been found to have low levels of phyla Firmicutes, Proteobacteria, and Actinobacteria, and the bacterial family *Lactobacillaceae* (5). Although, *Bacteroides* are abundant in children with celiac disease compared with healthy individuals while *Bifidobacterium* is lower regardless of dietary gluten consumption (4), it remains unknown whether the change in gut microbiota composition is a consequence of an already established intestinal inflammation or if genetically predisposed individuals have different bacterial colonization that increase the propensity to develop celiac disease.

Probiotics have varying mechanisms of action depending on species (6) and are known to influence the host health in three main ways: by modulating the existing microbiota, by communicating with the intestinal mucosa, and/or by affecting functions that are not limited to the gastrointestinal tract, such as the immune system. Supplementation of the probiotic bacteria, *Bifidobacterium breve* B632, restored some of the main microbial components in adults with celiac disease (5). However, there is a paucity of studies on the effects of probiotics in children with celiac disease, and those that have been performed have mostly been studying the effect of *Bifidobacterium* supplements. *Lactobacillus* has proven effects on the intestinal permeability and the immune system and may counteract translocation and inhibit the growth of harmful bacteria (7–10). In a previous study, the present investigators showed that a mixture of *Lactiplantibacillus plantarum* HEAL9 and *Lacticaseibacillus paracasei* 8700:2 had a dampening effect of peripheral immune response in children with ongoing celiac disease autoimmunity (CDA) (11).

An incongruence has previously been found in the effect after probiotic consumption by patients with celiac disease. The reasons for contradictory results might be multifactorial. First, the beta-diversity and microbiota composition differ in individuals with different HLA genotypes (2, 3). Second, gut microbiota continues to develop during the first 3 years of life; and thereafter, it has only minor fluctuations in the composition which may be affected by diet, a concomitantly occurring gut disease, treatment with antibiotics, age, and geographical location (12, 13). Finally, no double-blind placebo-controlled study has previously been performed to study the effects of probiotics on gut diversity in children with CDA on a normal gluten-containing diet.

This study aimed to analyze the effects of 6 months of supplementation with *L. plantarum* HEAL9 and *L. paracasei* 8700:2 on the gut microbiota composition and possible correlation between the gut microbiota composition and change in tissue transglutaminase autoantibody (tTGA) levels over time serving as a proxy for ongoing CDA in children at-risk for celiac disease.

## Materials and Methods

### Study Participants

Celiac Disease Prevention with Probiotics (CiPP) study is a double-blind placebo-controlled trial described in detail elsewhere (11). A total of 118 children with CDA, herein defined as having two consecutive samples with elevated levels of tTGA > 1.3 U/ml taken at least 3 months apart (14), were invited to participate in CiPP of whom 89 accepted participation and 78 children completed a 6-month follow-up of either receiving a mixture of *L. plantarum* HEAL9 and *L. paracasei* 8700:2 (probiotic group) or placebo (placebo group) (Table 1). The study population has been described in detail elsewhere (11). Briefly, the children were between 3 and 7 years of age (probiotic group: median age 5 (range 3–7) years; placebo group: median age 4 (range 3–6) years, *p* = 0.284). At the start of the study, median levels of IgA-tTG and IgG-tTG levels were 4.7 (range 1.6–12.2) U/ml and 1.6 (range 1.1–4.7) U/ml in the probiotic group, respectively, compared with 4.4 (range 1.9–13.2) U/ml and 1.6 (1.2–4.6) U/ml in the placebo group (*p* = 0.848 and *p* = 0.891), respectively. There was no difference in the distribution of HLA risk-haplotypes between the two groups: DR3-DQ2/DR3-DQ2 (*n* = 15 in the probiotic group and *n* = 13 in the placebo group), DR3-DQ2/DR4-DQ8 (*n* = 10 in the probiotic group and *n* = 16 in the placebo group), DR4-DQ8/DR4-DQ8 (*n* = 10 in the probiotic group and *n* = 7 in the placebo group), DR4-DQ8/DR4-DQ8 (*n* = 4 in the probiotic group and *n* = 2 in the placebo group), and DR4/DR12 (*n* = 1 in the probiotic group and *n* = 0 in the placebo group).

**Table 1:** Relative abundance of different phyla in study participants either receiving a mixture of *Lactiplantibacillus plantarum* HEAL9 and *Lacticaseibacillus paracasei* 8700:2 (Probiotics) or maltodextrin (Placebo) at baseline and after 6 months of intervention.

**Phyla**	**Probiotics 0 months (mean ± SD%)**	**Placebo 0 months (mean ± SD %)**	***p*-value**	**Probiotics 6 months (mean ± SD %)**	**Placebo 6 months (mean ± SD %)**	***p*-value**	***p*-value over time (Probiotics)**	***p*-value over time (Placebo)**	**Probiotics 6–0 months (mean ± SD %)**	**Placebo 6–0 months (mean ± SD %)**	***p*-value difference between groups**
Actinobacteria	0.9 ± 1.0	0.7 ± 0.6	0.24	0.8 ± 1.6	0.4 ± 0.3	0.49	0.049	0.07	−0.2 ± 1.7	−0.3 ± 0.7	0.80
Bacteroidetes	38.1 ± 19.5	43.6 ± 19.9	0.21	45.7 ± 18.7	50.8 ± 14.5	0.49	0.06	0.22	7.3 ± 22.8	8.8 ± 22.3	0.87
Firmicutes	55.9 ± 17.3	52.2 ± 19.7	0.38	49.5 ± 18.3	45.7 ± 15.0	0.24	0.10	0.22	−6.8 ± 22.7	−5.2 ± 21.8	0.74
Fusobacteria	0.003 ± 0.01	0.004 ± 0.008	0.34	0.003 ± 0.02	0.002 ± 0.006	0.93	0.33	0.05	0.0 ± 0.02	−0.0 ± 0.01	0.79
Lentisphaerae	0.003 ± 0.01	0.0006 ± 0.004	0.12	0.003 ± 0.02	0.00 ± 0.00	0.17	0.29	0.34	0.0 ± 0.01	0.0 ± 0.0	0.66
Proteobacteria	3.3 ± 6.5	1.6 ± 2.3	0.92	1.8 ± 2.7	1.8 ± 2.7	0.72	0.89	0.68	−0.9 ± 5.2	0.2 ± 3.5	0.95
Synergistetes	0.0007 ± 0.004	0.00 ± 0.00	0.35	0.00 ± 0.00	0.00 ± 0.00	NA	0.34	NA	0.0 ± 0.004	0.0 ± 0.0	0.34
Tenericutes	0.0004 ± 0.002	0.01 ± 0.07	0.96	0.0003 ± 0.002	0.0002 ± 0.001	1.00	1.00	1.00	0.0 ± 0.0	−0.01 ± 0.07	0.97
TM7	0.00 ± 0.00	0.00 ± 0.0004	0.31	0.00 ± 0.00	0.00 ± 0.00	NA	NA	0.34	0.0 ± 0.0	0.0 ± 0.0	0.32
Verrucomicrobia	1.7 ± 2.8	2.0 ± 3.6	0.63	2.3 ± 5.3	1.3 ± 1.9	0.70	0.76	0.54	0.5 ± 5.3	−0.7 ± 3.8	0.83

After randomization, 40 children received a 10^10^ cfu/g 1:1 mixture of *L*. [former *Lactobacillus* (15)] *plantarum* HEAL9 (DSM 15312) and *L*. [former *Lactobacillus* (15)] *paracasei* 8700:2 (DSM 13434) (probiotic group) and 38 children received 1 g maltodextrin (Glucidex IT-19, Roquette, Lesterrand, France) and yeast peptone (HYP-A, BioSpringer, Maisons-Alfort, France) (placebo group) daily for 6 months. The study product was provided in capsules, and the guardian of the child was instructed to mix the powder with cold food or fruit. Study participants were instructed to continue a gluten-containing diet and exclude other food products containing probiotics. During the intervention, six children in the probiotic group and four in the placebo group reported having taken antibiotics (*p* = 0.561). Stool samples and blood samples were collected at baseline and after 3 and 6 months of intervention. All samples were stored at −80°C prior to analysis. The study was approved by the Ethics Committee of the Medical Faculty, Lund University, on September 8, 2011 (Dnr 2011/335), and the study is registered in ClinicalTrials.gov (NCT03176095).

### DNA Extraction

DNA was extracted from the frozen fecal samples using an extraction method previously described (16). Briefly, 50 mg of the samples were mixed with 1 ml of sterile PBS buffer and incubated for 10 min prior to a bead beating on an Eppendorf Mixer (model 5432, Eppendorf, Hamburg, Germany) at 4°C for 30 min. The samples were centrifuged at 13,000 rpm for 1 min and about, 200 ml of the supernatant was transferred to a clean 2.0 ml sample tube. DNA was extracted from the supernatant using the EZ1 DNA extraction robot (Qiagen bioinformatics, Aarhus, Denmark) and the DNA tissue kit (Qiagen bioinformatics, Aarhus, Denmark). The extracted DNA was frozen at −20°C until further analysis.

### Terminal Restriction Fragment Length Polymorphism

The 16S rRNA genes were amplified using the universal primer FAM-ENV1 (5′-AGA GTT TGA TII TGG CTC AG-3′, fluorescently labeled with FAM dye at the 5′end) and ENV2 (5′-CGG ITA CCT TGT TAC GAC TT-3′) as previously described (17). The PCR product were run through a 1.5% (w/v) agarose gel in TAE-buffer (VWR, Radnor, Pennsylvania) to verify the reaction after staining the gel with red (VWR, Radnor, Pennsylvania). The PCR product was purified with a MinElute PCR purification kit (Qiagen bioinformatics, Aarhus, Denmark) according to instructions from the manufacturer. The DNA was eluted by using 10 μl of EB buffer, and the concentration of the eluate was determined with NanoDrop ND-1000 (Saveen Werner, Limhamn, Sweden) at 285 nm. Two hundred nanograms of the resulting DNA product were digested with the restriction endonuclease *Msp*I fast digest (Thermo Fisher scientific, Sweden) according to instructions from the manufacturer. The products were diluted 5 times with nuclease-free water (Qiagen bioinformatics, Aarhus, Denmark) in a sterile PCR plate with 96 wells (Sarstedt, Numbrecht, Germany) and analyzed on an ABI 3130xl Genetic analyzer (Applied Biosystems, USA) with GeneScan LIZ 600 (range 20–600 bases, Applied Biosystems) as internal size standard at DNA-lab (SUS, Malmö, Sweden). The T-RFLP data was analyzed with GeneMapper® software version 4.1 (Applied Biosystems). A local southern algorithm was chosen for size calling, and the size range was set from 40 to 600 base pairs. The relative area percentage was calculated for each T-RF and used for calculations of Shannon and Simpson's diversity indices.

### 16S rRNA Gene Sequencing

The variable region V3–V4 of the 16S rRNA gene was amplified according to the 16S metagenomic sequencing library preparation protocol provided by Illumina but using the primers 341F with underlined Illumina adapter overhang (5′-TCG GCA GCG TCA GAT GTG TAT AAG AGA CAG CCT ACG GGN GGC WGC AG-3′) and 805R with underlined Illumina adapter overhang (5′-GTC TCG TGG GCT CGG AGA TGT GTA TAA GAG ACA GGA CTA CHV GGG TAT CTA ATC C-3′) (18) (Eurofin genomics, Ebersberg, Germany), generating an amplicon length of 550 bp. The thermal cycling was performed in an Eppendorf MasterCycler (Eppendorf, Hamburg, Germany). The amplified PCR products were purified using AMPure XP beads (Agencourt, Beckman coulter genomics). Indexes (Nextera XT index kit, Illumina) were attached to the PCR product in a second PCR reaction and the amplified PCR products were purified one more time. Concentrations of DNA were determined using Qbit4.0 Fluorometer (ThermoFisher scientific, Sweden), and samples were combined in equimolar ratios to a final concentration of 4 pM before being sequenced on an Illumina MiSeq (Illumina, USA) by using MiSeq reagent kit v3 (Illumina Inc., San Diego, USA) with a read length of 2 × 300 bp paired-end sequencing according to the instructions from the manufacturer. Both the forward and reversed obtained sequences were trimmed at 25 and 275 bp. After filtering, a total of 11,815,795 sequence reads was obtained and the mean number of reads per sample was 52,052. The data were analyzed using the pipeline QIIME2 and processed through several R software packages, such as vegan and phyloseq. The resulting sequences were filtered to include only bacterial reads on domain level and to exclude mitochondria and chloroplasts. Alpha diversity was determined as Simpson and Shannon's indices based on non-rarefied data. Beta diversity was calculated using the weighted and unweighted UniFrac, Jaccard, and Bray-Curtis dissimilarity. Closed referenced OTU picking and taxonomic assignment were performed with Greengenes database (99.9% identity, v 13.5), which is based on the former taxonomic classification of *Lactobacillaceae* as described by Winslow et al. [(19) (Approved Lists 1980)]. The amplicon sequence variants (ASV:s) that significantly changed within a group were determined using DESeq2 with a significant level at 0.001.

### Radioligand Binding Assays

Plasma IgA-tTG and IgG-tTG levels were determined at baseline and after 3 and 6 months as previously described (11).

### Statistics

T-RFLP results were analyzed using the software Sigma Plot 12.0 (Systat software, Inc.). The Mann-Whitney rank-sum test was used to compare differences between the two groups for diversity indices and T-RFs abundance. Changes in bacterial relative abundance between the different groups were determined using R version 3.5.1 (20) and the non-parametric Wilcoxon-rank-sum test on total sum scaled data. Wilcoxon-signed-rank-sum test was carried out to compare relative abundance over time within each group. Wilcoxon-rank-sum test was also used to determine differences in alpha diversity between the groups over time. Differences in beta diversity were calculated using the Adonis method. Pairwise correlations between the gut microbiota and autoantibody levels were analyzed using the corr.test function in the psych R package, which computed Spearman's correlations and adjusted with the Holm method. Prior to the analysis, the gut microbiota was agglomerated at the genus level, and only the genus present in all samples were taken into consideration.

## Results

### Study Participants

As reported previously (11), there was no difference between the groups in IgA-tTG or IgG-tTG levels after 3 months (*p* = 0.362 and *p* = 0.925, respectively) or after 6 months (*p* = 0.838 and *p* = 0.766, respectively). Compared with baseline, IgA-tTG levels decreased a median 0.85 (IQR −3.30–0.24) U/ml (*p* = 0.013), while IgG-tTG decreased a median 0.29 (IQR −1.31–0.40) U/ml (*p* = 0.062) after 6 months in the probiotic group. During the same time, IgA-tTG decreased a median 0.79 (IQR −3.43–0.08) U/ml (*p* = 0.043) and IgG-tTG decreased a median 0.36 (IQR −1.12–0.05) U/ml (*p* = 0.008) in the placebo group. During the intervention period, three children from the probiotic group and four children from the placebo group reported symptoms such as pain, flatulence, or diarrhea, and one child in each group had gastrointestinal symptoms. Study compliance was followed up by questionnaires and that the study participants returned empty packages of the study product.

### Alpha and Beta Diversity

**Figure 1:**
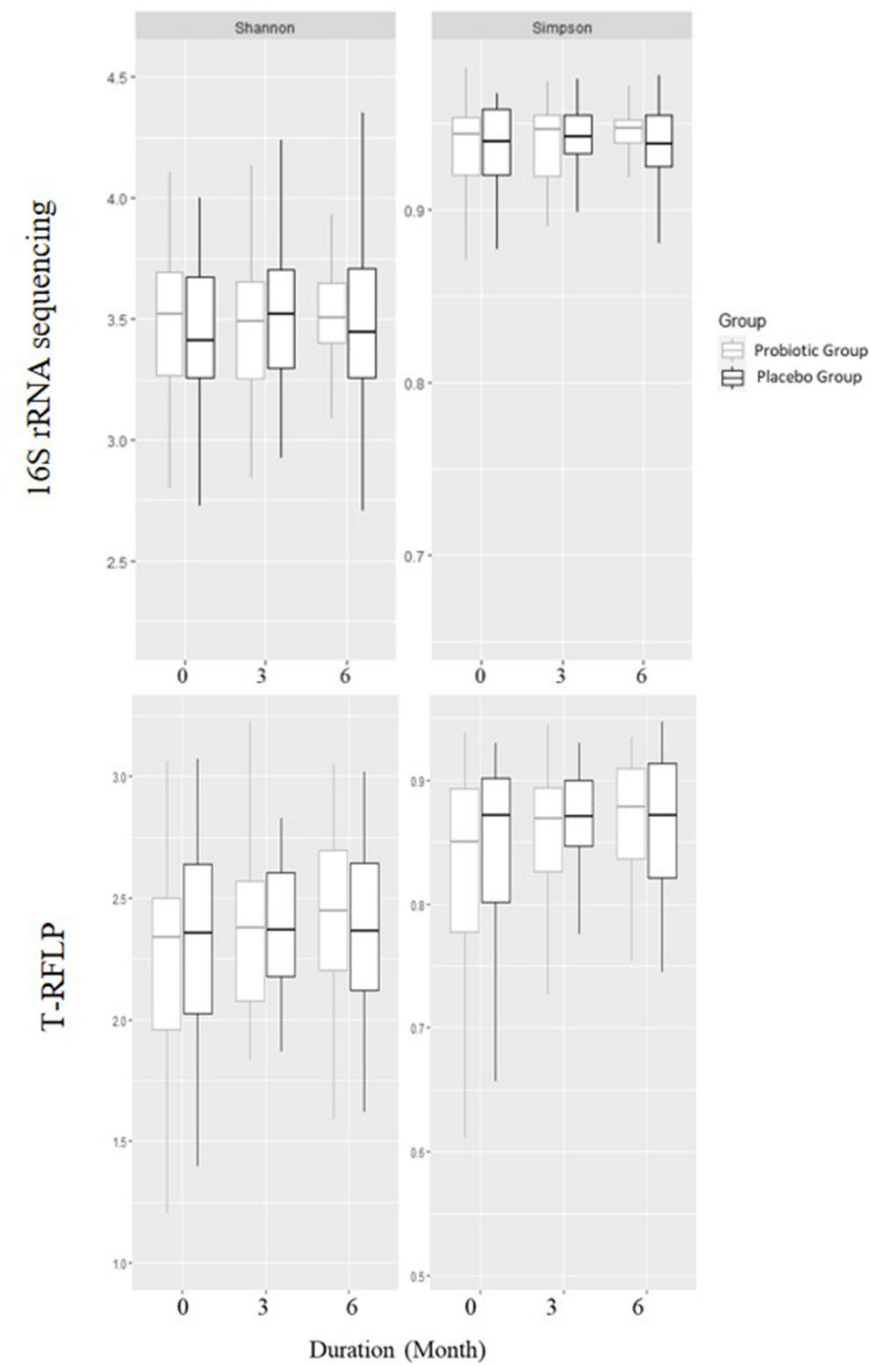
Alpha diversities using Shannon and Simpson's diversity indexes based on 16S rRNA gene sequencing and T-RFLP in children receiving either a mixture of *Lactiplantibacillus plantarum* HEAL9 and *Lacticaseibacillus paracasei* 8700:2 (probiotic group) or maltodextrin (placebo group).

There was no difference in alpha diversity measures between the two groups or within each group (Figure 1) at the start of the study or after 3 and 6 months.

Furthermore, there was no difference between the groups in beta diversity calculated withdistance measure at any time point. The beta diversity remained constant over the study period, while studying the groups independently.

### Changes in the Relative Abundance of Bacterial Taxa Over Time

The sequencing discovered 10 different phyla in the data set. At the start of the study, the highest prevalence was found for the two phyla, Firmicutes and Bacteroidetes followed by Proteobacteria, Actinobacteria, and Verrucomicrobia (Table 1).

The relative abundance of Actinobacteria decreased over time in the probiotic group (*p* < 0.05), whereas, not in the placebo group (*p* = 0.07) (Table 1). Furthermore, the relative abundance of Fusobacteria decreased slightly in the placebo group over time (*p* = 0.05). On a family level, bacteria belonging to the *Lactocacillaceae* increased in the probiotic group (*p* < 0.0001) (Table 2). There was also a difference in the relative abundance of *Lactobacillaceae* between the two groups after 6 months of intervention, where the probiotic group had a higher relative abundance compared with the placebo group (*p* < 0.0001). In the probiotic group, the relative abundance of *Bifidobacteriaceae*, furthermore, decreased from a mean of 0.9 ± 0.9% to a mean of 0.7 ± 1.5% (*p* = 0.048). A decrease in *Streptococcaceae* from a mean of 0.2 ± 0.5% to a mean of 0.07 ± 0.1 % (*p* = 0.01) was found in the placebo group.

**Table 2:** Relative abundance of different families in study participants either receiving a mixture of *L. plantarum* HEAL9 and *L. paracasei* 8700:2 (Probiotics) or maltodextrin (Placebo) at baseline and after 6 months of intervention.

**Family**	**Probiotics 0 months (mean ± SD%)**	**Placebo 0 months (mean ± SD%)**	***p*-value**	**Probiotics 6 months (mean ± SD%)**	**Placebo 6 months (mean ± SD %)**	***p*-value**	***p*-value over time (Probiotics)**	***p*-value over time (Placebo)**	**Probiotics 6–0 months (mean ± SD%)**	**Placebo 6–0 months (mean ± SD%)**	***p*-value difference between groups**
*[Barnesiellaceae]*	1.1 ± 1.8	1.6 ± 2.3	0.25	1.2 ± 1.8	1.9 ± 2.5	0.37	0.44	0.38	0.1 ± 1.9	0.4 ± 2.6	0.52
*[Mogibacteriaceae]*	0.1 ± 0.1	0.1 ± 0.2	0.19	0.1 ± 0.1	0.07 ± 0.06	0.16	0.25	0.33	−0.04 ± 0.1	−0.04 ± 0.1	0.47
*[Odoribacteraceae]*	0.2 ± 0.4	0.2 ± 0.2	0.83	0.4 ± 0.5	0.4 ± 0.6	0.97	0.32	0.23	0.1 ± 0.4	0.2 ± 0.5	0.88
*Alcaligenaceae*	0.4 ± 0.8	0.3 ± 0.4	0.84	0.4 ± 0.5	0.4 ± 0.5	0.88	0.61	0.61	0.04 ± 0.5	0.1 ± 0.5	0.67
*Bacteroidaceae*	26.4 ± 17.8	28.2 ± 1.8	0.64	29.7 ± 15.0	32.6 ± 13.9	0.61	0.26	0.23	3.3 ± 15.8	5.4 ± 15.7	0.30
*Bifidobacteriaceae*	0.9 ± 0.9	0.5 ± 0.5	0.17	0.7 ± 1.5	0.3 ± 0.3	0.41	0.048	0.06	−0.2 ± 1.6	−0.3 ± 0.6	0.84
*Christensenellaceae*	0.2 ± 0.6	0.1 ± 0.2	0.88	0.2 ± 0.3	0.08 ± 0.01	0.35	0.78	0.55	−0.1 ± 0.4	0.0 ± 0.1	0.73
*Clostridiaceae*	2.5 ± 5.4	1.0 ± 1.3	0.35	1.7 ± 3.2	0.9 ± 1.6	0.29	0.70	0.56	−0.5 ± 5.9	−0.2 ± 2.1	0.91
*Coriobacteriaceae*	0.08 ± 0.1	0.1 ± 0.2	0.68	0.07 ± 0.09	0.07 ± 0.1	0.99	0.34	0.58	−0.01 ± 0.1	−0.04 ± 0.2	0.80
*Enterobacteriaceae*	1.7 ± 4.4	0.6 ± 1.2	0.70	1.0 ± 2.4	0.7 ± 2.4	0.21	0.51	0.29	−0.2 ± 3.1	0.1 ± 2.8	0.44
*Erysipelotrichaceae*	0.4 ± 0.5	0.7 ± 1.5	0.89	0.5 ± 1.0	0.3 ± 0.7	0.50	0.56	0.17	0.1 ± 0.8	−0.4 ± 1.0	0.13
*Lachnospiraceae*	13.2 ± 6.3	12.4 ± 9.8	0.11	12.8 ± 9.4	12.9 ± 8.5	0.95	0.26	0.53	−0.7 ± 10.2	0.6 ± 11.1	0.19
*Lactobacillaceae*	0.02 ± 0.07	0.02 ± 0.08	0.43	0.09 ± 0.2	0.002 ± 0.007	<0.0001	<0.0001	0.09	0.1 ± 0.2	−0.02 ± 0.08	<0.0001
*Pasteurellaceae*	1.2 ± 3.3	0.6 ± 1.6	0.97	0.3 ± 0.5	0.7 ± 1.2	0.87	0.39	0.74	−0.8 ± 3.3	0.03 ± 1.8	0.49
*Porphyromonadaceae*	2.0 ± 5.2	2.3 ± 2.4	0.51	2.4 ± 3.1	3.1 ± 3.0	0.11	0.65	0.16	0.5 ± 3.1	0.8 ± 3.2	0.40
*Prevotellaceae*	1.9 ± 5.2	4.5 ± 10.3	0.14	5.1 ± 13.0	4.4 ± 9.9	0.76	0.94	0.31	3.1 ± 11.4	−0.2 ± 9.3	0.09
*Rikenellaceae*	6.3 ± 6.9	6.7 ± 6.6	0.69	6.3 ± 5.2	8.1 ± 6.2	0.24	0.50	0.22	−0.02 ± 6.9	2.0 ± 8.5	0.33
*Ruminococcaceae*	33.4 ± 13.0	30.4 ± 13.8	0.20	28.7 ± 12.3	26.4 ± 7.8	0.73	0.08	0.40	−5.1 ± 13.2	−2.9 ± 13.3	0.59
*Streptococcaceae*	0.3 ± 0.5	0.2 ± 0.5	1.00	0.2 ± 0.7	0.07 ± 0.1	0.08	0.40	0.01	0.0 ± 0.8	−0.2 ± 0.5	0.15
*Turicibacteraceae*	0.5 ± 1.0	0.4 ± 1.3	0.14	0.4 ± 1.2	0.2 ± 0.6	0.61	0.12	0.44	−0.05 ± 1.4	−0.1 ± 1.4	0.29
*Veillonellaceae*	5.1 ± 4.5	6.9 ± 6.8	0.39	4.7 ± 4.4	4.8 ± 5.6	0.70	0.67	0.20	−0.5 ± 5.2	−2.0 ± 7.6	0.35
*Verrucomicrobiaceae*	1.7 ± 2.8	2.0 ± 3.6	0.63	2.3 ± 5.3	1.3 ± 1.9	0.70	0.76	0.54	0.5 ± 5.4	−0.7 ± 3.8	0.83

The relative abundance of the former genus *Lactobacillus* increased in the probiotic group (*p* < 0.001), while it decreased in the placebo group (*p* = 0.01), which also differed between the two groups after 6 months of intervention (*p* < 0.0001). Furthermore, there was a decrease in relative abundance for both *Lactococcus* and *Bifidobacterium* in the probiotic group (*p* < 0.05), whereas, the opposite was true for *Streptococcus* which remained constant in the probiotic group but decreased in the placebo group (*p* < 0.01) (Table 3).

**Table 3:** Relative abundance of the different genus in study participants either receiving a mixture of *L. plantarum* HEAL9 and *L. paracasei* 8700:2 (Probiotics) or maltodextrin (Placebo) at baseline and after 6 months of intervention.

**Genus**	**Probiotics 0 months (mean ± SD%)**	**Placebo 0 months (mean ± SD%)**	***p*-value**	**Probiotics 6 months (mean ± SD%)**	**Placebo 6 months (mean ± SD%)**	***p*-value**	***p*-value over time (Probiotics)**	***p*-value over time (Placebo)**	**Probiotics 6–0 months (mean ± SD%)**	**Placebo 6–0 months (mean ± SD %)**	***p*-value difference between groups**
*Akkermansia*	1.7 ± 2.8	2.0 ± 3.6	0.63	2.3 ± 5.3	1.3 ± 1.9	0.70	0.76	0.54	0.5 ± 5.4	−0.7 ± 3.8	0.83
*Anaerostipes*	0.2 ± 0.4	0.2 ± 0.3	0.03	0.4 ± 1.1	0.1 ± 0.3	0.22	0.94	0.36	0.2 ± 1.1	−0.03 ± 0.2	0.85
*Bacteroides*	26.4 ± 17.8	28.2 ± 17.6	0.64	29.7 ± 15.0	32.6 ± 13.9	0.61	0.26	0.23	3.3 ± 15.8	5.4 ± 15.7	0.30
*Bifidobacterium*	0.9 ± 0.9	0.5 ± 0.5	0.17	0.7 ± 1.5	0.3 ± 0.3	0.41	0.048	0.06	−0.2 ± 1.6	−0.3 ± 0.6	0.84
*Blautia*	0.6 ± 0.8	0.4 ± 0.5	0.03	0.6 ± 0.7	0.5 ± 0.7	0.28	0.82	0.56	0.0 ± 1.0	0.05 ± 0.7	0.44
*Clostridium*	0.2 ± 0.2	0.2 ± 0.3	1.00	0.2 ± 0.4	0.3 ± 0.3	0.61	0.96	0.59	0.1 ± 0.4	0.04 ± 0.3	0.54
*Collinsella*	0.05 ± 0.1	0.04 ± 0.1	0.36	0.03 ± 0.07	0.03 ± 0.07	0.77	0.28	0.98	−0.02 ± 0.1	−0.01 ± 0.1	0.25
*Coprococcus*	1.5 ± 1.5	1.0 ± 1.1	0.27	1.1 ± 0.8	1.1 ± 0.9	0.86	0.56	0.79	−0.5 ± 1.6	0.1 ± 1.3	0.21
*Dialister*	4.0 ± 4.5	5.5 ± 6.9	0.65	4.1 ± 4.4	4.3 ± 5.8	0.55	0.74	0.62	−0.04 ± 5.2	−1.2 ± 7.3	0.48
*Dorea*	0.9 ± 1.5	0.8 ± 1.4	0.90	0.7 ± 1.5	0.6 ± 1.3	0.84	0.41	0.33	−0.3 ± 1.7	−0.2 ± 2.0	0.62
*Faecalibacterium*	18.1 ± 11.4	17.2 ± 10.5	0.81	16.4 ± 9.4	15.2 ± 8.3	0.51	0.72	0.52	−2.0 ± 13.9	−1.2 ± 9.3	0.94
*Haemophilus*	1.1 ± 3.2	0.6 ± 1.6	1.00	0.3 ± 0.5	0.7 ± 1.2	0.83	0.40	0.74	−0.7 ± 3.2	0.03 ± 1.7	0.49
*Holdemania*	0.08 ± 0.2	0.05 ± 0.08	0.60	0.06 ± 0.1	0.04 ± 0.05	0.59	0.91	0.96	−0.03 ± 0.1	−0.01 ± 0.1	0.68
*Lachnobacterium*	0.2 ± 0.6	0.1 ± 0.4	0.81	0.2 ± 0.6	0.2 ± 0.6	0.07	0.50	0.23	−0.1 ± 0.9	0.1 ± 0.8	0.12
*Lachnospira*	0.9 ± 1.0	0.6 ± 0.9	0.06	0.7 ± 0.9	0.6 ± 1.1	0.37	0.41	0.76	−0.2 ± 1.3	0.1 ± 1.4	0.37
*Lactobacillus*	0.01 ± 0.06	0.01 ± 0.05	0.26	0.05 ± 0.1	0.00 ± 0.00	<0.0001	<0.001	0.01	0.05 ± 0.1	−0.01 ± 0.05	<0.0001
*Lactococcus*	0.04 ± 0.02	0.0008 ± 0.004	0.10	0.0005 ± 0.003	0.002 ± 0.01	0.54	0.04	0.97	−0.04 ± 0.2	0.0 ± 0.01	0.12
*Odoribacter*	0.2 ± 0.3	0.1 ± 0.2	0.47	0.3 ± 0.4	0.3 ± 0.5	0.81	0.29	0.18	0.1 ± 0.4	0.2 ± 0.5	0.67
*Oscillospira*	3.1 ± 3.4	2.4 ± 0.3	0.41	2.8 ± 2.7	2.4 ± 1.9	0.92	0.91	0.49	−0.4 ± 3.3	0.2 ± 2.3	0.36
*Parabacteroides*	1.8 ± 1.9	2.3 ± 2.4	0.36	2.4 ± 3.1	3.1 ± 3.0	0.08	0.59	0.14	0.6 ± 2.8	0.8 ± 3.1	0.40
*Prevotella*	1.9 ± 5.2	4.5 ± 10.3	0.14	5.1 ± 13.0	4.4 ± 9.9	0.76	0.94	0.31	3.1 ± 11.4	−0.2 ± 9.3	0.09
*Roseburia*	3.6 ± 2.9	3.5 ± 4.3	0.25	5.0 ± 7.0	5.0 ± 7.1	0.62	0.77	0.56	1.3 ± 6.9	1.4 ± 7.0	0.60
*Ruminococcus*	3.5 ± 4.2	2.3 ± 2.4	0.16	3.0 ± 2.8	2.2 ± 2.4	0.20	0.56	0.78	−0.5 ± 4.2	−0.04 ± 2.0	0.67
*SMB53*	0.1 ± 0.2	0.07 ± 0.2	0.32	0.2 ± 0.3	0.07 ± 0.1	0.87	0.43	0.70	0.1 ± 0.2	0.0 ± 0.1	0.68
*Streptococcus*	0.3 ± 0.5	0.2 ± 0.5	0.94	0.2 ± 0.7	0.07 ± 0.1	0.06	0.46	0.007	0.05 ± 0.7	−0.2 ± 0.5	0.13
*Sutterella*	0.4 ± 0.8	0.3 ± 0.4	0.84	0.4 ± 0.5	0.4 ± 0.5	0.88	0.61	0.61	0.04 ± 0.5	0.1 ± 0.5	0.67
*Turicibacter*	0.5 ± 1.0	0.4 ± 1.3	0.14	0.4 ± 1.2	0.2 ± 0.6	0.61	0.12	0.44	−0.05 ± 1.4	−0.1 ± 1.4	0.29
*Veillonella*	0.4 ± 1.1	1.1 ± 3.0	0.75	0.2 ± 0.4	0.4 ± 0.8	0.57	0.80	1.00	−0.2 ± 0.9	−0.7 ± 2.9	0.45

### Changes in ASV:s Abundance Over Time

**Figure 2:**
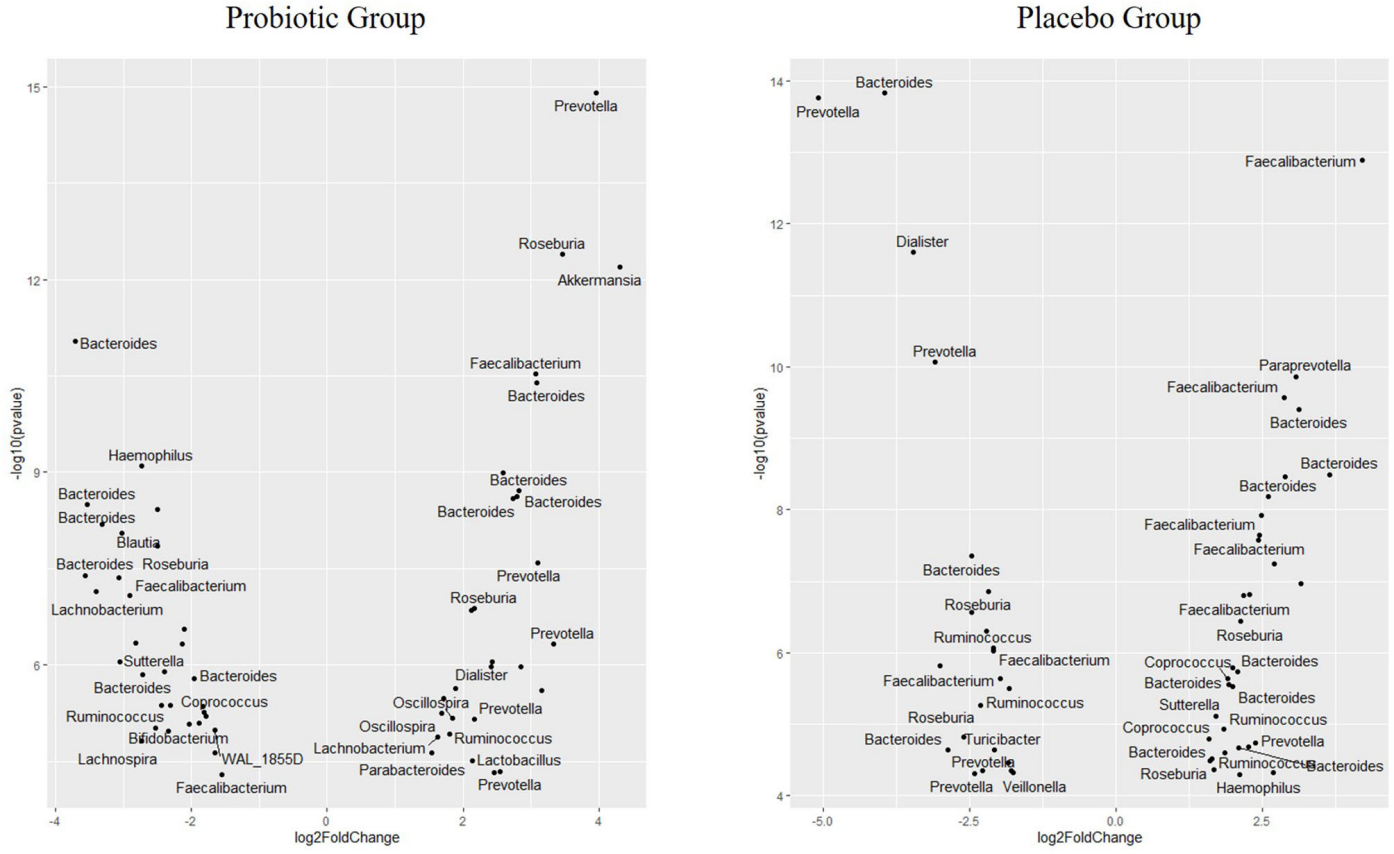
Volcano plot showing differences in the abundance of specific microbial ASV in children receiving either a mixture of *L. plantarum* HEAL9 and *L. paracasei* 8700:2 (probiotic group) or maltodextrin (placebo group) expressed as Log2 Fold change (end of study/start of study). All ASV:s in the figure changed significantly over time with *p* < 0.001.

When analyzing different abundances of ASV:s using Deseq2, 59 ASV:s were changed in the probiotic group (all with *p* < 0.001), while 56 ASV:s were changed in the placebo group (all with *p* < 0.001). Furthermore, differing ASV:s belonging to a higher diversity of genus were found in the probiotic group (*n* = 16 genera) compared with the placebo group (*n* = 12 genera). The abundance of ASV:s belonging to the genera *Akkermansia* increased in the probiotic group, while *Bifidobacterium* decreased in the probiotic group (Figure 2). Only one ASV:s belonging to *Lactobacillus* increased in abundance in the probiotic group. Similar changes were observed for all significant ASV:s belonging to *Prevotella*, which increased in the probiotic group but mainly decreased in the placebo group (Figure 2). More ASV:s belonging to *Bacteroides* were decreased in the probiotic group compared with the placebo group. The ASV:s belonging to *Coprococcus* increased in the placebo group while they decreased in the probiotic group (Figure 2). Furthermore, the abundance of ASV:s belonging to *Dialister, Haemophilus*, and *Sutterella* changes in different directions in the two respective groups. In the probiotic group, the abundance of ASV:s belonging to *Oscillospira* and *Parabacteroides* increased while *Blautia, Lachnospira*, and *Sutterella* decreased (Figure 2). In the placebo group, the abundance of ASV:s were decreased for *Dialister, Turicibacter*, and *Veillonella* while *Paraprevotella* were increased (Figure 2).

### Correlation Between Autoantibody Levels and Gut Microbiota on Genus Level

**Figure 3:**
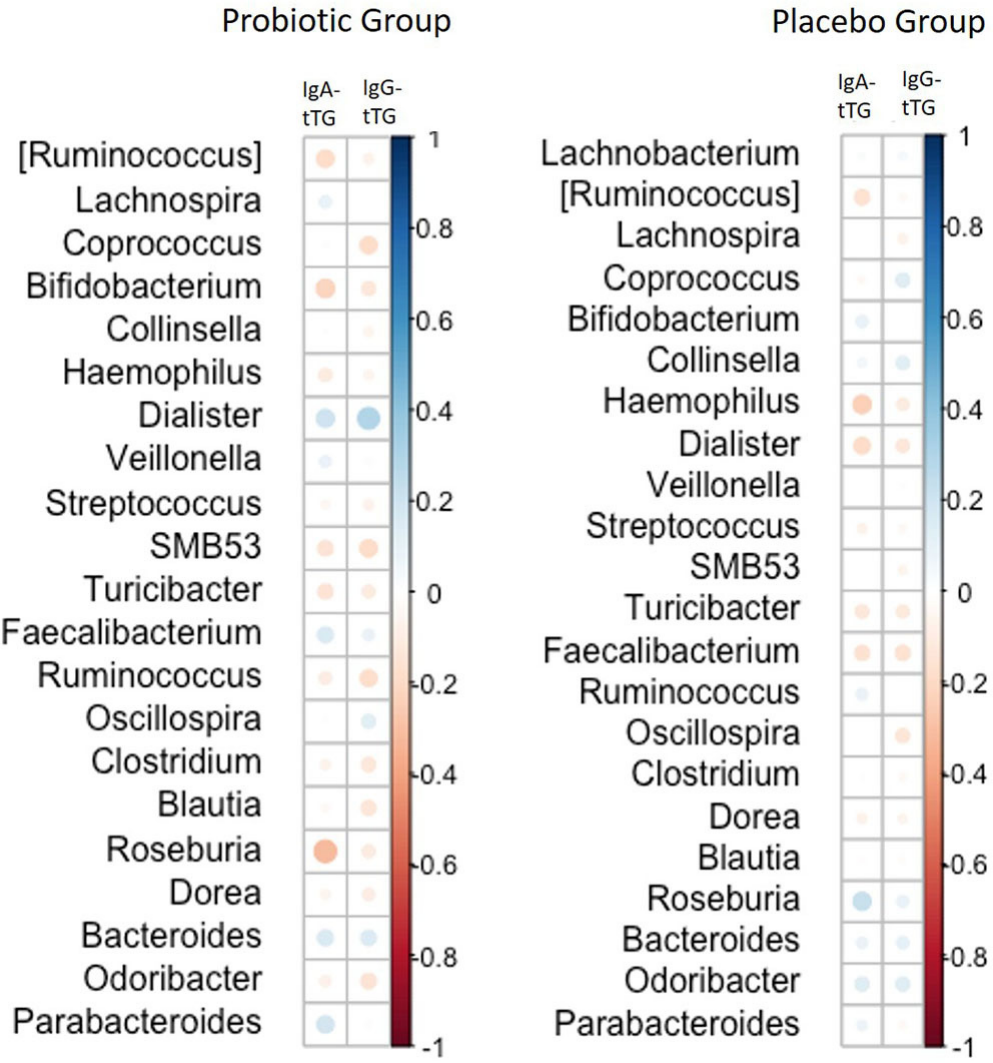
Correlations between the amount of gut microbiota on genus level and levels of IgA-tTG and IgG-tTG in children receiving either a mixture of *L. plantarum* HEAL9 and *L. paracasei* 8700:2 (probiotic group) or maltodextrin (placebo group). Positive correlations are marked in blue circles and inverse correlations in red circles. The size of the circles corresponds to the magnitude of Spearman's rho correlation.

There was a correlation between the amount of microbiota on the genus level for *Dialister* and IgG-tTG levels during the intervention period observed in the probiotic group (rho = 0.35, *p* < 0.01) (Figure 3). However, no such correlation was found for IgA-tTG antibodies and no correlations between *Dialister* and IgG-tTG or IgA-tTG were detected in the placebo group.

## Discussion

This study found that the relative abundance of *Lactobacillus* increased as expected over time in children with CDA receiving probiotic supplementation containing *L. plantarum* HEAL9 and *L. paracasei* 8700:2 compared with children with CDA receiving placebo. There was no change in alpha or beta diversities between or within the two groups over time. However, several differences were found on an ASV level, suggesting a change in different directions in bacterial composition by an intervention of *L. plantarum* HEAL9 and *L. paracasei* 8700:2.

Although, probiotics have proven effects on the improvement of gastrointestinal symptoms in several studies (21–23), there are only few reports on probiotic supplementation that changes alpha and beta diversity measures. In the present study, the alpha and beta diversity remained constant regardless of receiving probiotics or not, indicating that the overall microbiota is relatively stable in children with CDA over time. Similar results regarding alpha diversity were observed in a previous study after investigating the effects of *Bifidobacterium breve* for 3 months in children with celiac disease (5). In line with our results, another study found no differences in the alpha and beta diversities in patients with celiac disease compared with healthy controls (24). Although, it is known that beta diversity differs between risk groups depending on the types of HLA (3, 25) and could explain the differences in study population, the present study did not have enough power to do separate sub-analyses stratified by HLA genotype. However, the diversity measures indicated that the intestinal microbiota was relatively stable in the study participants who received probiotics or not.

Changes in gut microbiota composition after probiotic consumption have been reported in children with already treated celiac disease. In addition, the phyla Proteobacteria has been reported to be lower in children with already treated celiac disease as compared with healthy controls (4). Although, the present study did not observe an overall change in this phylum in children with CDA, the abundance of ASV:s belonging to *Haemophilus* and *Sutterella* increased in the placebo group and decreased in the probiotic group. Furthermore, *Prevotella* increased among the children that received probiotics for 6 months, while it mainly decreased in the placebo group. *Prevotella* is a genus in the phyla Bacteroides, whose concentration has found to be correlated with inflammation. Interestingly, celiac disease has been proposed to be driven by a Th-17 immune response (26), and *Prevotella* species have induced a similar response in autoimmune arthritis (25).

Another genus, *Akkermansia*, is lower in relative number in patients with celiac disease compared with healthy controls (27). In the present study, the ASV abundance of *Akkermansia* increased in the probiotic group over time. Although, *Akkermansia* is increased in type 2 diabetes, it is generally considered to be associated with gastrointestinal health (28). Indeed, both ASV:s belonging to *Prevotella* and *Akkermansia* have been found in higher abundance in healthy controls compared with patients with celiac disease (24), which contradicts the observations that *Prevotella* correlates with inflammation in autoimmune arthritis (25). The genus *Prevotella* has a high genetic diversity both between and within species, which could explain discrepancies in correlations between certain diseases and the abundance of *Prevotella* (29). In the present study, increasing abundances of ASV:s belonging to both genus *Prevotella* and *Akkermansia* in the probiotic group were observed, which may indicate that *Lactobacillus* may restore species belonging to those genera to normal levels.

In children with celiac disease, *Bacteroides* were increased, whereas, bacteria belonging to the genera *Lactobacillus* and *Bifidobacterium* were decreased compared with healthy individuals (4). Although, the relative abundance of *Bacteroides* remained unchanged in the present study, the results from the analyses of ASV abundance indicated that individual bacteria in this genus changed in CDA children over time. This is in accordance with a previous study observing differences in *Bacteroides fragilis* between patients with celiac disease and healthy controls (5). In the present study, the relative abundance of *Bifidobacterium* decreased in the probiotic group during an intervention, which was also reflected by a decreasing abundance of ASV belonging to this genus. It has been reported that *Bifidobacterium* naturally declines with age (12), but it is unlikely that only 6 months of intervention is causing the observed decline.

Celiac disease is known to primarily affect the upper part of the intestine, which is also the main habitat of *Bifidobacterium*. *Lactobacillaceae* species also primarily colonize this part of the intestine. It is plausible that the decrease in *Bifidobacterium* is due to a change in the microbiome composition in the upper intestinal tract.

Although, *Lactobacillus* species constitute only a small proportion of the overall fecal microbiota and the observed difference was minor when considering the relative number, the increase in the *Lactobacillus* genus that was observed only in the probiotic group indicates excellent compliance among the study participants. The genus *Lactobacillus* may have been found in higher amounts in the small intestine since it has been demonstrated that more changes in bacterial composition are expected to be found in duodenal biopsies compared with fecal samples (24). This could explain why only few changes in the overall fecal microbiota composition were observed in our study, both reflected by the relative abundance and diversity measures, respectively, although, another study indicated that there are similarities between the fecal microbiota and the bacterial composition in small intestinal biopsies in celiac disease (4).

Another finding from the present study was the decrease in the relative abundance of *Streptococcus* in the placebo group compared with the probiotic group. *Streptococcus* is known to produce enzymes involved in gluten degradation (30), and a decrease in this genus might indicate a less efficient gluten metabolism and that a higher proportion of immunogenic gluten peptides is present in the gut. *Streptococcus* is known to be a major constituent of the microbiota in the mouth and the upper gastrointestinal tract, and presumably, the effect of the probiotics on those microbial communities is much more pronounced than the effects seen in fecal samples. A previous study has shown that gluten can be degraded by *Lactobacillus* spp. *in vitro* (31), which would suggest that the decrease in the tTG levels observed over time in the probiotic group could be caused by a more efficient gluten metabolism due to higher abundance of *lactobacilli*. However, a similar decrease was seen in the placebo group, which contradicts this theory.

The effects of *L. plantarum* HEAL9 and *L. paracasei* 8700:2 on the study endpoints, CDA and celiac disease, have already been published elsewhere (11). In the present study, these previous results were extended with analysis on correlations of the microbiome composition with tTGA levels over time. A correlation was only found for the abundance of *Dialister* with higher levels of IgG-tTG in the probiotic group, indicating differences between the two groups regarding immunological response to gut microbiota. *Dialister invisus* has previously been found in elevated levels in children with islet autoimmunity at risk for type 1 diabetes and associated with increased gut permeability (32). This could indicate that a higher abundance of *Dialister* leads to an increased gut permeability, which reflect the increase in IgG-tTG levels. However, this was not observed for IgA-tTG, which could be explained by the faster response of those autoantibodies to changes in the intestinal environment as previously described (33).

In conclusion, a mixture of *L. plantarum* HEAL9 and *L. paracasei* 8700:2 have no major effects ondiversity indexes but may have pronounced effects on ASV levels after 6 months of intervention. This indicates that specific bacteria are affected by the intake of this specific probiotic mixture in children with CDA. Larger studies are warranted to further study the long-term effects of probiotics in preventing celiac disease in at-risk children.

## Data Availability Statement

The data presented in the study are deposited in the NCBI repository, accession number PRJNA732664.

## Ethics Statement

The studies involving human participants were reviewed and approved by Ethics Committee of the Medical Faculty, Lund University. Written informed consent to participate in this study was provided by the participants' legal guardian/next of kin.

## Author Contributions

EO drafted the manuscript, carried out the statistical analysis, interpreted the data, and completed all subsequent revisions until submission. ÅH, CA, and GM interpreted the data and reviewed and revised the manuscript. CA coordinated the collection of the samples and data collection from study participants. DA was the principal investigator for the study and responsible for study design, conceptualized the study, advised in the presentation of analysis results, interpreted the data, reviewed, and revised the manuscript, critically, for important intellectual content. All authors contributed to the article and approved the submitted version.

## Conflict of Interest

DA is stated as an inventor in a patent application based on the results of the clinical trial but has signed over all legal rights to the patent to Probi AB. Probi AB has developed and supplied the study material (active and placebo products) for the trial as well as financially supported the trial with minor costs for analyzing material. None of the authors are employed by Probi AB and no salaries, consultancy fees, etc., have been paid by Probi AB to the authors in connection with the trial. GM is a minority shareholder in Probi AB. The remaining authors declare that the research was conducted in the absence of any commercial or financial relationships that could be construed as a potential conflict of interest.
